# Delayed hemolysis, elevated liver enzymes, low platelet count syndrome in succession of switches of preventive anticoagulant treatment in a 41-year-old patient with a history of recurrent assisted implantation failures: a case report

**DOI:** 10.1186/s13256-018-1943-1

**Published:** 2019-01-19

**Authors:** Katrin Mikolaiczik, Marc Praetner, Michael Rüth, Karlheinz Mark

**Affiliations:** 1 Rottal-Inn-Kliniken Krankenhaus Eggenfelden, Simonsöder Allee 20, 84307 Eggenfelden, Germany; 2 Walter Brendel Centre for Experimental Medicine, Ludwig-Maximilians-Universität München, Marchioninistraße 15, 81377 Munich, Germany; 3 Kliniken Nordoberpfalz AG, Krankenhaus Tirschenreuth, St.-Peter-Str. 31, 95643 Tirschenreuth, Germany; 4 Kliniken Nordoberpfalz AG, Klinikum Weiden, Söllnerstraße 16, 92637 Weiden, Germany

**Keywords:** HELLP syndrome, Preeclampsia, Low molecular weight heparin, Aspirin, ICSI, IVF

## Abstract

**Background:**

For the past decades the mean age of primiparae in Western societies is constantly increasing. At the same time, there is a growing demand for assisted reproductive technologies such as *in vitro* fertilization and intracytoplasmic sperm injection. Subsequently, a higher prevalence of pregnancy-associated diseases such as gestational hypertension and preeclampsia is observed. To improve pregnancy rates after *in vitro* fertilization/intracytoplasmic sperm injection and to reduce the risk of pregnancy-associated diseases with a cardiovascular pathophysiology, two anticoagulants are the focus of current research: low molecular weight heparin and acetylsalicylic acid (aspirin).

**Case presentation:**

A 41-year-old white woman, gravida 3, para 0, received low molecular weight heparin to reduce the risk of abortion after five unsuccessful intracytoplasmic sperm injections and two miscarriages. She autonomously discontinued the medication with low molecular weight heparin at 12 weeks and 2 days of gestation and took aspirin instead until 24 weeks and 2 days of gestation as preeclampsia prophylaxis. However, the pregnancy ended with an urgent cesarean section at 27 weeks and 4 days of gestation due to a fast progressing hemolysis, elevated liver enzyme levels, and low blood platelet count syndrome, a potentially life-threatening variant of preeclampsia.

**Conclusion:**

Based on the current demographic trend toward late-in-life pregnancy it is mandatory to establish clear guidelines concerning preventive treatment options of preeclampsia for patients with risk factors. The establishment of a special first-trimester screening for these women should be discussed. Moreover, it is necessary to raise the awareness among physicians of these contemporary issues to guarantee the best possible medical care.

**Electronic supplementary material:**

The online version of this article (https://doi.org/10.1186/s13256-018-1943-1) contains supplementary material, which is available to authorized users.

## Background

In Western societies the mean age of women giving birth to their first child is constantly increasing [1, 2]. This epidemiological trend is accompanied by higher rates of infertility, preterm delivery, and a rising incidence of pregnancy-associated diseases such as maternal diabetes, chronic hypertension, and preeclampsia [3–5]. At the same time there is a growing demand for assisted reproductive technologies such as *in vitro* fertilizations (IVFs) and intracytoplasmic sperm injections (ICSIs) [4, 6]. Therefore, current clinical research focuses not only on improving pregnancy rates after IVF/ICSI but also on reducing the risks of developing pregnancy-associated complications. As increased thrombin generation, inflammation, and vascular lesions are regarded as the pathophysiological hallmarks of preeclampsia and recurrent abortions, two anticoagulants are currently in use as preventive treatments: low molecular weight heparin (LMWH) and acetylsalicylic acid (aspirin) [7].

Whereas most data do not support a beneficial effect of aspirin on pregnancy rates after IVF/ICSI, the application of LMWH seems to improve birth rates, particularly after recurrent implantation failures [8–12]. Regarding preeclampsia, the prophylactic use of aspirin is discussed ambivalently in the literature. However, the latest clinical data suggest that patients with higher risk factors, such as history of preeclampsia, seem to benefit from aspirin, especially when the therapy is initiated in early pregnancy [13–16]. LMWH alone as well as in combination with aspirin seems to be able to reduce the prevalence of preeclampsia [17–21]. Despite these positive findings, a recent meta-analysis from 2016 was not able to detect a beneficial effect of LMWH on recurrent placental-mediated complications such as preeclampsia in women at risk [22]. In summary, the existing data do not allow us to draw final clinical conclusions due to the mixed results reported in the current literature.

In this report the case of a 41-year-old patient will be discussed; she was treated with LMWH for the first fortnight after ICSI. She continued the treatment with LMWH until 12 weeks and 2 days of gestation and then decided to take aspirin instead until 24 weeks and 2 days of gestation. Based on her own literature research she decided to adjust her medication autonomously to reduce the risks of another abortion or preeclampsia. The pregnancy ended with an urgent cesarean section (C-section) at 27 weeks and 4 days of gestation due to fast progressing hemolysis, elevated liver enzyme levels, and low blood platelet count (HELLP) syndrome, a potentially life-threatening variant of preeclampsia characterized by the core symptoms: hemolysis, elevated liver enzymes, and low platelets [23].

## Case presentation

A 41-year-old white woman, gravida 3, para 0, was admitted to our clinic at 27 weeks and 3 days of gestation. She reported suffering from dizziness, impaired vision, facial edema as well as increasing edema in her lower legs since the previous day. She also mentioned raised blood pressure (approximately 175/105 mmHg) although regularly taking her medication of alpha-methyl-dopa 250 mg 1-2-1. We initiated this therapy 3 weeks earlier due to the development of gestational hypertension. Furthermore, she took magnesium 40 mg 1-1-1 and progesterone 100 mg 2-0-2 since the onset of pregnancy as supportive medication. She had no other additional medication. She had no history of pre-existing diseases. Pregnancy-associated complications never occurred in her family.

Obstetric history: At the age of 38, after 3 years of trying to get pregnant, she decided on assisted reproductive technologies. She had three inseminations, followed by five ICSIs without success. The sixth ICSI finally led to pregnancy, although ending with an early abortion at 6 weeks of gestation. After the seventh ICSI two embryos were transferred. In addition, LMWH was prescribed for the first 14 days after transfer. In the following ultrasound examinations only one viable embryo could be detected. However, this pregnancy ended at 7 weeks of gestation. After the second miscarriage our patient and her husband ran through genetic counselling and testing, revealing no pathologies. Furthermore, antiphospholipid syndrome (APS), lupus erythematosus, and thrombophilia were excluded. In search of other possible reasons explaining the dissatisfying clinical course, our patient once more had an ultrasound of the genital organs now revealing a tumor at the posterior wall of her uterus, most probably representing a fibroma. The following hysteroscopy showed an arcuate uterus without the need to interfere surgically.

Then, our patient, now 41-years old, went for another ICSI with the transfer of two embryos. Initially, follow-up ultrasounds showed two amniotic cavities with viable embryos. At 8 weeks of gestation one embryo died and could no longer be detected in the subsequent ultrasound examinations (“vanishing twin”). Again, LMWH (nadroparin 0.4 ml/day subcutaneous injection) was prescribed for the first 14 days after transfer. Based on literature research and out of fear of another miscarriage our patient decided to continue the treatment with nadroparin. Although discussing this topic with her gynecologist, she could not receive the prescription as the gynecologist expressed a lack of experience in preventive anticoagulant treatment after ICSI. Our patient therefore turned to her general practitioner who issued the prescription for nadroparin. With 12 weeks and 2 days of gestation she autonomously discontinued the treatment with nadroparin and changed to aspirin 100 mg/day instead as preeclampsia prophylaxis. At 21 weeks and 2 days of gestation a prenatal care ultrasound revealed bilateral uterine artery notching. Three weeks later she developed hypertensive blood pressure values leading to hospitalization in our clinic. We initiated an antihypertensive therapy with alpha-methyl-dopa 250 mg as described above. The medication with aspirin was discontinued. To estimate further clinical course, preeclampsia-associated angiogenesis biomarkers were analyzed: The soluble fms-like tyrosine kinase-1 (sFlt-1), an antiangiogenic factor, and the placental growth factor (PlGF), an angiogenic factor. Although the cause of preeclampsia is not yet fully understood, angiogenic imbalance in favor of antiangiogenic factors like sFlt-1 is involved in the pathophysiology of preeclampsia resulting in abnormal remodeling of maternal spiral arteries which finally leads to placental malperfusion [24, 25]. The sFlt-1/PIGF ratio already increases before the onset of the clinical symptoms and is therefore used as a predictor for preeclampsia with high sensitive and specific values [24–27]. Her sFlt-1/PIGF ratio of 276 (physiological sFlt/PIGF ratio < 33) indicated a high risk of developing preeclampsia in the next 4 weeks. We therefore initiated a glucocorticoid prophylaxis with betamethasone for respiratory distress syndrome in case of preterm delivery. After adjusting her blood pressure to 150/80–100 mmHg our patient was discharged after 7 days.

Finally, at 27 weeks and 3 days of gestation, she again was admitted to our clinic, now with the symptoms described above leading to the diagnosis of preeclampsia. Cardiotocography (CTG) results and obstetric ultrasound were normal apart from an elevated pulsatility index of 1.2 of the umbilical artery Doppler. Bilateral uterine artery notching could still be detected. Our patient’s blood work did not show any abnormalities apart from recently elevated lactate dehydrogenase (LDH) values (see Additional file 1: Table S1). Blood samples were now taken in a close-meshed timescale: within 16 hours transaminases and hemolysis parameters were rapidly increasing and thrombocyte counts were decreasing, thus fulfilling the criteria of a fast progressing HELLP syndrome (see Additional file 1: Table S1).

An urgent C-section was performed based on maternal indication due to the deteriorating clinical condition of our patient: constantly raised blood pressure > 185/105 mmHg despite additional treatment with nifedipine; growing pain in her upper abdomen; oliguria with increasing leg, arm, and facial edema; beginning somnolence and apathy. At 27 weeks and 4 days of gestation a girl was delivered, weighing 873 g, with Apgar scores of 8/10/10, umbilical cord pH = 7.35. The girl was immediately attended by the department of pediatrics. The uterus *in situ* appeared to show signs of diffuse myohyperplasia. In addition, several smaller pedunculated uterine fibroids were apparent. Histopathology of the placenta revealed premature ripened chorionic villi and an older circumscribed infarction.

After the C-section our patient was transferred to the intensive care unit (ICU). Postoperative sonography revealed small pleural effusions in both lungs. Diuretic therapy with furosemide rapidly increased urinary excretion. Additional antihypertensive medication with urapidil via syringe pump infusion could be reduced gradually and terminated after 2 days. Perception disorders such as illusory conjunctions in the perception of objects rapidly disappeared. After 2 days she could be transferred to our general ward. She recovered quickly, her blood work normalized. Her blood pressure was adjusted to 140–159/90–99 mmHg with alpha-methyl-dopa 250 mg 1-0-1. Eight days after the C-section she was able to leave our hospital. We recommended follow-up blood samples and regular blood pressure measurements for the next 6 weeks. Antihypertensive medication should be adjusted if applicable. Furthermore, we suggested 24-hour blood pressure measurements on an annual basis due to the higher prevalence of cardiovascular diseases after preeclampsia [28, 29].

Apart from a pneumothorax in combination with respiratory distress syndrome within the first days the child did not suffer from any severe complications during her hospital stay. With a weight of 2680 g the healthy girl was finally discharged after 77 days.

## Discussion and conclusion

LMWH is discussed to have a positive influence on birth rates after recurrent implantation failures [10]. After five unsuccessful ICSIs and one miscarriage our patient received LMWH for the first time. However, two following LMWH-supported pregnancies ended in miscarriage. It can be debated whether she would have had benefited from an application of LMWH after the third unsuccessful ICSI. As among other possible reasons for her recurrent implantation failures and infertility, there are the observed uterine abnormalities: the clinically apparent myohyperplasia which is often caused by endometriosis, as well as the uterine fibroids and the arcuate uterus [30–32]. These anatomical anomalies alone have a negative effect on pregnancy rates and are not directly affected by LMWH.

Regarding preeclampsia, our patient had the following risk factors: advanced age (> 40), nulliparity, and ICSI [4, 5, 33, 34]. The early twin pregnancy could have had an adverse effect as well, as multiple pregnancy is known to correlate positively with the incidence of preeclampsia [5]. However, current data suggest that the vanishing twin phenomenon is associated with small for gestational age babies, preterm delivery, and low birth weight but not preeclampsia [35]. The circumscribed infarction of the placenta could be a remnant of the vanishing twin. On the other hand, casual pathologies of the development of preeclampsia are occlusive hypertensive lesions in the spiral arteries and inadequate placentation which lead to placental ischemia and finally to infarction of the placenta [35–39].

Bilateral arterial notching and elevated sFlt-1/PlGF ratio are pregnancy-associated risk factors for the development of preeclampsia [40–44]. With 21 weeks and 2 days of gestation, bilateral arterial notching was detected by umbilical artery Doppler assessment and 3 weeks later, after the development of gestational hypertension, we detected the elevated sFlt-1/PlGF ratio. In synopsis of the pregnancy-associated and anamnestic risk factors, the sFlt-1/PlGF ratio could have been calculated when the bilateral notching was detected at 21 weeks and 2 days of gestation. However, at that time our patient had already taken aspirin autonomously which is the recommended preventive medication of preeclampsia. When we diagnosed the gestational hypertension at 24 weeks of gestation we discontinued this treatment. This decision could be discussed as on the one hand she had not had preeclampsia yet and still faced the increasing probability of developing one. On the other hand she had already shown clear signs of pathophysiological changes, such as the bilateral arterial notching and finally gestational hypertension. This finally led to the conclusion that aspirin as preventive treatment was of no further use. Unfortunately, there are no current data regarding the recommendation of continuing or quitting the medication of aspirin after developing gestational hypertension. A meta-analysis from 2010, however, indicated that low-dose aspirin started at 16 weeks or earlier is not only associated with a reduction in severe preeclampsia and gestational hypertension but also with a reduction in preterm birth [15]. Our patient therefore might have benefited from a continuous application of aspirin. In conclusion, a continuous application of LMWH and aspirin after transfer might have been the best preventive treatment for our patient.

Due to the current demographic trend more patients will face an elevated probability of pregnancy-associated diseases such as preeclampsia. Therefore, there is a need to establish first-trimester screening and a definitive guideline for preventive treatment of preeclampsia in women with risk factors, especially if the case is more complex like the one presented. Moreover, our patient did not receive sufficient medical advice from her pretreating gynecologists concerning the possibilities of improving the birth rate after recurrent implantation failures and preventing the development of preeclampsia. As a result she felt impelled to start a preventive medication on her own based on literature research. Thus, there is not only the need for a guideline but also the need of raising awareness among gynecologists of these contemporary issues.

## Additional file

Additional file 1: **Table S1.** Blood sample values since admission. *ALT* alanine transaminase, *AST* aspartate transaminase, *LDH* Lactate dehydrogenase. (DOCX 20 kb)

## Abbreviations

APS: Antiphospholipid syndrome
Aspirin: Acetylsalicylic acid
C-section: Cesarean section
CTG: Cardiotocography
HELLP: Hemolysis, elevated liver enzyme levels, and low blood platelet count
ICSI: Intracytoplasmic sperm injection
ICU: Intensive care unit
IVF: *In vitro* fertilization
LDH: Lactate dehydrogenase
LMWH: Low molecular weight heparin
PlGF: Placental growth factor
sFlt-1: Soluble fms-like tyrosine kinase-1
