# Supplementary material for: Delayed hemolysis, elevated liver enzymes, low platelet count syndrome in succession of switches of preventive anticoagulant treatment in a 41-year-old patient with a history of recurrent assisted implantation failures: a case report
Source: J Med Case Rep. 2019 Jan 19;13:16. doi: 10.1186/s13256-018-1943-1 (PMC6339357; doi:10.1186/s13256-018-1943-1)
Supplement: Supplementary file 1 — Table S1. Blood sample values since admission. ALT alanine transaminase, AST aspartate transaminase, LDH Lactate dehydrogenase. (DOCX 20 kb) [file 13256_2018_1943_MOESM1_ESM.docx]

**Supplement “Delayed HELLP syndrome in succession of preventive anticoagulant treatment-switch in a 41-year old patient with a history of recurrent assisted implantation failures: a case report.”**

*Table 1: Blood sample values since admission*

|  | Limit values | Admission | 6 h after admission | 12 h after admission | 16 h after admission |
| --- | --- | --- | --- | --- | --- |
| AST | 10-35 U/L | 30 | 95 | 391 | 483 |
| ALT | 10-35 U/L | 19 | 61 | 232 | 279 |
| Thrombocytes | 150-370 /nl | 264 | 248 | 148 | 138 |
| Haptoglobin | 30-200 mg/dl | 110 | 109 | <30 | <30 |
| LDH | 0-247 U/L | 292 | 334 | 767 | 1063 |
| Bilirubin | 0,0- 1,0 mg/dl | 0,3 | 0,4 | 1,0 | 1,4 |
